# Diagnosis and clinical management of hepatosplenic schistosomiasis: A scoping review of the literature

**DOI:** 10.1371/journal.pntd.0009191

**Published:** 2021-03-25

**Authors:** Francesca Tamarozzi, Veronica A. Fittipaldo, Hans Martin Orth, Joachim Richter, Dora Buonfrate, Niccolò Riccardi, Federico G. Gobbi

**Affiliations:** 1 Department of Infectious-Tropical Diseases and Microbiology, IRCCS (Istituto di Ricovero e Cura a Carattere Scientifico) Sacro Cuore Don Calabria Hospital, Negrar di Valpolicella, Verona, Italy; 2 Department of Gastroenterology, Hepatology and Infectious Diseases, Duesseldorf University Hospital, Heinrich Heine University, Düsseldorf, Germany; 3 Institute of Tropical Medicine and International Health, Charité Universitätsmedizin, Berlin, Germany; Centers for Disease Control and Prevention, UNITED STATES

## Abstract

**Background:**

Hepatosplenic schistosomiasis (HSS) is a disease caused by chronic infection with *Schistosma* spp. parasites residing in the mesenteric plexus; portal hypertension causing gastrointestinal bleeding is the most dangerous complication of this condition. HSS requires complex clinical management, but no specific guidelines exist. We aimed to provide a comprehensive picture of consolidated findings and knowledge gaps on the diagnosis and treatment of HSS.

**Methodology/principal findings:**

We reviewed relevant original publications including patients with HSS with no coinfections, published in the past 40 years, identified through MEDLINE and EMBASE databases. Treatment with praziquantel and HSS-associated pulmonary hypertension were not investigated. Of the included 60 publications, 13 focused on diagnostic aspects, 45 on therapeutic aspects, and 2 on both aspects. Results were summarized using effect direction plots. The most common diagnostic approaches to stratify patients based on the risk of variceal bleeding included the use of ultrasonography and platelet counts; on the contrary, evaluation and use of noninvasive tools to guide the choice of therapeutic interventions are lacking. Publications on therapeutic aspects included treatment with beta-blockers, local management of esophageal varices, surgical procedures, and transjugular intrahepatic portosystemic shunt. Overall, treatment approaches and measured outcomes were heterogeneous, and data on interventions for primary prevention of gastrointestinal bleeding and on the long-term follow-up after interventions were lacking.

**Conclusions:**

Most interventions have been developed on the basis of individual groups’ experiences and almost never rigorously compared; furthermore, there is a lack of data regarding which parameters can guide the choice of intervention. These results highlight a dramatic need for the implementation of rigorous prospective studies with long-term follow-up in different settings to fill such fundamental gaps, still present for a disease affecting millions of patients worldwide.

## Author summary

Hepatosplenic schistosomiasis (HSS) is a complex clinical condition caused by the consequence of current or past chronic infection with *Schistosma* spp. parasites residing in the mesenteric veins. HSS encompasses a characteristic type of portal fibrosis, portal hypertension, and derived complications, the most dangerous of which being gastrointestinal bleeding. It has been estimated that 0.2 million deaths occur every year in sub-Saharan Africa due to this complication. HSS requires a complex clinical management, but no specific guidelines exist. We aimed to provide a comprehensive picture of consolidated findings and knowledge gaps, by reviewing the scientific literature published in the past 40 years on the diagnosis and treatment of HSS. Of the included 60 publications, 13 focused on diagnostic aspects, 45 on therapeutic aspects (including treatment with beta-blockers, local management of esophageal varices, surgical procedures, and transjugular intrahepatic portosystemic shunt), and 2 on both aspects. Overall, treatment approaches and measured outcomes were heterogeneous; furthermore, there is a lack of data regarding which parameters should guide the choice of intervention and on the long-term outcome of interventions. These results highlight a dramatic need for the implementation of rigorous prospective studies to fill fundamental gaps, still present for a disease affecting millions of patients worldwide.

## Introduction

Schistosomiasis is the disease caused by the infection with trematode parasites of the genus *Schistosoma*. The main species causing intestinal and hepatosplenic disease are *Schistosoma mansoni*, distributed in sub-Saharan Africa and Latin America, mainly Brazil, and *Schistosoma japonicum* and *Schistosoma mekongi* in East Asia, mainly in China and the Philippines [1]. According to recent estimates, 440 million people suffer from chronic schistosomiasis, about one-third of whom suffer from either current or the consequences of past infection with *S*. *mansoni* [2–4]. Outside endemic areas, about 20% of migrants from endemic areas may be infected [5].

**Fig 1:**
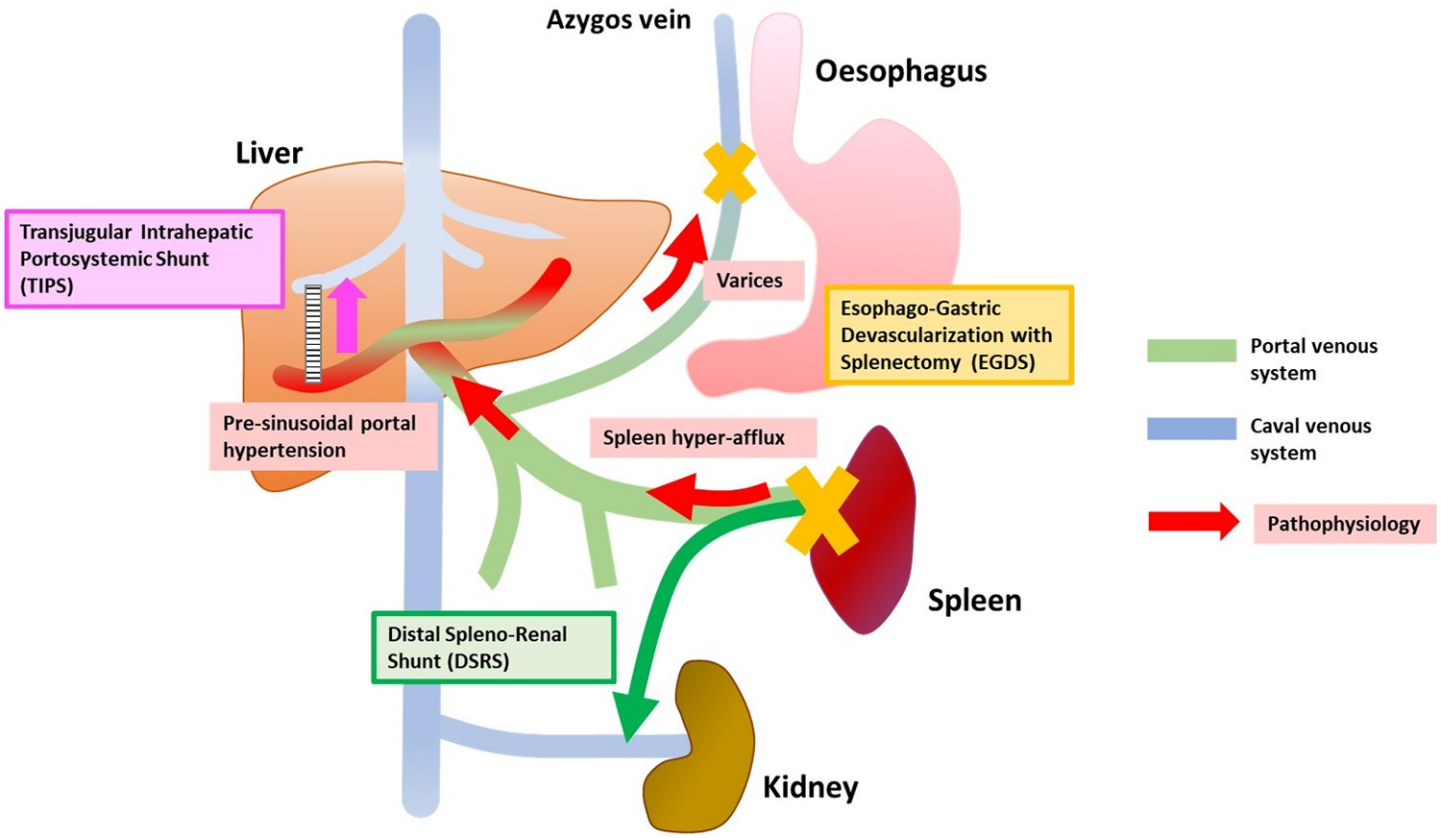
Schematic representation of the main pathological mechanisms of HSS (red arrows and boxes) and of the main surgical approaches (Edged colored boxes). The chronic granulomatous reaction around *Schistosoma* spp. eggs embolized in the liver eventually results in presinusoidal portal hypertension, which in turn causes spleen congestion and the formation of varices. Portal hypertension is also supported by spleen hyperafflux. Hepatopetal portal flow is generally preserved. The “classical” surgical interventions for HSS include EGDS and DSRS. TIPS shunts the portal blood flow intrahepatically from the portal to the hepatic venous system. DSRS, distal splenorenal shunt; EGDS, esophagogastric devascularization procedure with splenectomy; HSS, hepatosplenic schistosomiasis; TIPS, transjugular intrahepatic portosystemic shunt.

People get infected through contact with freshwater contaminated with parasite larvae (cercariae). In the human host, the parasite matures into adults, which settle in the mesenteric venous plexuses. There, they excrete eggs through the intestinal wall via the host feces. However, a proportion of the released eggs are trapped in host tissues. Of these, most are retained in the intestinal wall, while some are transported by the mesenteric circulation to the liver, where they get trapped in the small portal branches. A granulomatous reaction forms around the eggs, causing small vessels obliteration, perivascular fibrosis, and intergranulomatous proliferation of new, abnormal vessels [6]. With the repeated embolization of eggs over time, especially in case of high-burden infection, this process involves increasingly larger portal trunks, progressing to presinusoidal portal hypertension and formation of portosystemic collateral veins [6,7]. Hepatosplenic schistosomiasis (HSS) encompasses a characteristic type of portal fibrosis, also called Symmer’s clay pipe fibrosis or periportal fibrosis (PPF), and its resulting complications (Fig 1), such as portal hypertension, splenomegaly, hypertensive gastropathy, portosystemic collaterals, esophageal varices, and upper gastrointestinal bleeding (UGB), which is the most dangerous complication of this condition [8,9]. It has been estimated that 0.2 million deaths occur every year in sub-Saharan Africa due to this complication [9]. UGB is estimated to occur in up to 80% of people with PPF, recurrent episodes being frequent, with a mortality rate per each bleeding episode of up to 30% [9].

As opposed to cirrhosis (Table 1), in HSS, the hepatic function is overall preserved [1]. Hematochemical alterations such as thrombocytopenia and leukopenia, classically attributed to “hypersplenism,” might be due to intrasplenic blood stasis, rather than sequestration with abnormal splenic function. Indeed, in HSS, these alterations are not accompanied by evident clinical manifestations and at least partially recover even after spleen-preserving interventions [10]. Increased liver enzymes, bilirubin, coagulation times and D-dimer levels, and reduced concentrations of coagulation factors, have been found to correlate with degree of PPF, and the lack of evident haemorrhagic diathesis has been attributed to the balance between reduced production of pro- and anticoagulation factors and increased levels of von Willebrand factor [11,12].

**Table 1:** Summary of the main different features of hepatosplenic schistosomiasis and cirrhosis.

Hepatosplenic schistosomiasis	Cirrhosis
• Confluent granulomata around trapped helminth ova → portal “pipe-stem” fibrosis around portal vessels; liver parenchyma preserved	• Liver cell necrosis followed by nodular hepatocellular regeneration and fibrous septa and bands involving the entire liver parenchyma
• Presinusoidal block; normal occluded hepatic venous pressure	• Sinusoidal block; increased occluded hepatic venous pressure
• Reactive splenic hyperplasia followed by congestive splenomegaly; overall splenohepatic blood hyperafflux	• Congestive splenomegaly; decreased splenohepatic blood flow
• Normal or increased hepatopetal portal flow	• Reduced hepatopetal or hepatofugal portal flow
• Liver function preserved	• Liver function impaired
• Thrombocytopenia + lack of evident hemorrhagic diathesis	• Thrombocytopenia + impaired coagulation function

El-Gendi and colleagues [13,14], using direct pressure measurements, described different haemodynamic patterns in patients with portal hypertension due to HSS, with variable predominance either of the presinusoidal hypertension or of the increased splenic flow (Fig 1). Overall, in HSS, hepatic flow is maintained in the normal range and a hyperdynamic systemic and splanchnic circulation occurs; hepatic artery flow regulation and increased blood flow from the spleen have been involved, although the exact pathophysiological mechanisms of such condition are not completely elucidated [6,7,15–17].

Antiparasitic treatment with praziquantel has been shown to interrupt the progression and to induce partial or complete regression of periportal fibrosis due to *S*. *mansoni* infection after months from administration, while effect on *S*. *japonicum*-induced fibrosis seems less evident [18,19]. The effect on portal hypertension may even precede that on fibrosis and occur in patients with unvaried liver fibrosis [19]. However, these effects are observed mostly in the presence of early-grade fibrosis and in patients of young age [18–23]. Therefore, praziquentel must be administered to all patients with schistosomiasis but is often not sufficient to control and treat the disease. Clinical management of HSS requires a complex approach aiming to reduce the risk of complications, most importantly variceal bleeding, and to decrease mortality; however, rigorous comparison between clinical approaches has been virtually never carried out, and no specific clinical management guidelines exist so far.

We reviewed the scientific literature published in the past 40 years on the diagnosis and treatment of HSS, with the aim of providing a comprehensive picture of consolidated findings and knowledge gaps, and define currently available clinical management strategies and future lines of clinical research.

## Methods

### Search strategy

The specific research questions addressed by this scoping review [24] were: (1) what are the diagnostic techniques useful for the routine detection and prognostic definition of complications of HSS (namely, portal hypertension, varices, and bleeding); and (2) what are the therapeutic approaches currently available for the prevention and/or elective treatment of the complications of HSS, namely, portal hypertension, varices characteristics, bleeding, and death. No formal review protocol was registered.

References were identified through MEDLINE (PubMed) and EMBASE databases, which were searched on March 13, 2020, using the strategy reported in S1 File. Articles were also identified through searches of the reference lists of reviews and relevant publications. No restriction was applied regarding language, publication type (full paper or conference report), and publication date, but only papers published from 1990 onward were eventually included in the review, to provide a picture of recent and current diagnosis and management of HSS. Search results were combined and duplicates removed before screening for relevance. The work is presented according to the recommendations of the PRISMA checklist for scoping reviews (S1 Table [25]).

### Inclusion and exclusion criteria, study selection, data extraction, and data synthesis

Original cross-sectional, cohort, case–control, and diagnostic accuracy studies, as well as clinical trials, were included in this review. Systematic reviews with meta-analyses were included if they presented data from potentially eligible studies not retrieved by the literature search and were in any case used to provide further data on investigated treatment comparisons. Case reports were included only when addressing new diagnostic or therapeutic interventions. Narrative reviews were excluded. Among studies with eligible design, only those for which data were extractable for patients with HSS with no coinfections were included. Studies were excluded if: (1) full text and abstract were both unavailable or only the abstract was available but did not convey the needed data; (2) human *Schistosoma* infection was not investigated or, if schistosomiasis was investigated, the focus was not HSS; (3) papers including patients coinfected with other hepatic diseases when data from the *Schistosoma*-only infected groups could not be extracted; (4) study duplication; and (5) narrative reviews. The diagnosis of schistosomiasis and the classification of the case as HSS were accepted as declared by the authors of the studies. Treatment with praziquantel and pulmonary hypertension complicating HSS were not investigated. For pulmonary hypertension, the reader can refer to recent publications [26,27].

Two authors (FT and NR) reviewed titles and abstracts of publications retrieved by the search to identify those potentially eligible. In case of doubt on the decision whether a study could be eligible, a third author (FG) reviewed the publication to reach a collegial decision. Data extracted were: (1) country where the study was conducted; (2) study design; (3) number of included patients with HSS and other relevant patients’ characteristics; (4) PPF grading; (5) diagnostic technique, for publications dealing with diagnostic aspects; (6) type of intervention(s), for publications dealing with clinical management; and (7) follow-up length, evaluated outcomes, and principal findings.

Data are presented as summaries of findings using Effect Direction Plots [28], grouped by paper focus (diagnostic or treatment) and by treatment category (pharmacological, local variceal management, classic surgical intervention, transjugular intrahepatic portosystemic shunt (TIPS), and transplant). No meta-analysis and formal quality assessment of extracted data was carried out; study type, effect direction of interventions on outcomes, differences between interventions or from baseline (together with statistical significance when reported), and sample size were visually plotted to provide an overall appraisal of the extracted data quality, characteristics, and heterogeneity.

## Results

**Fig 2:**
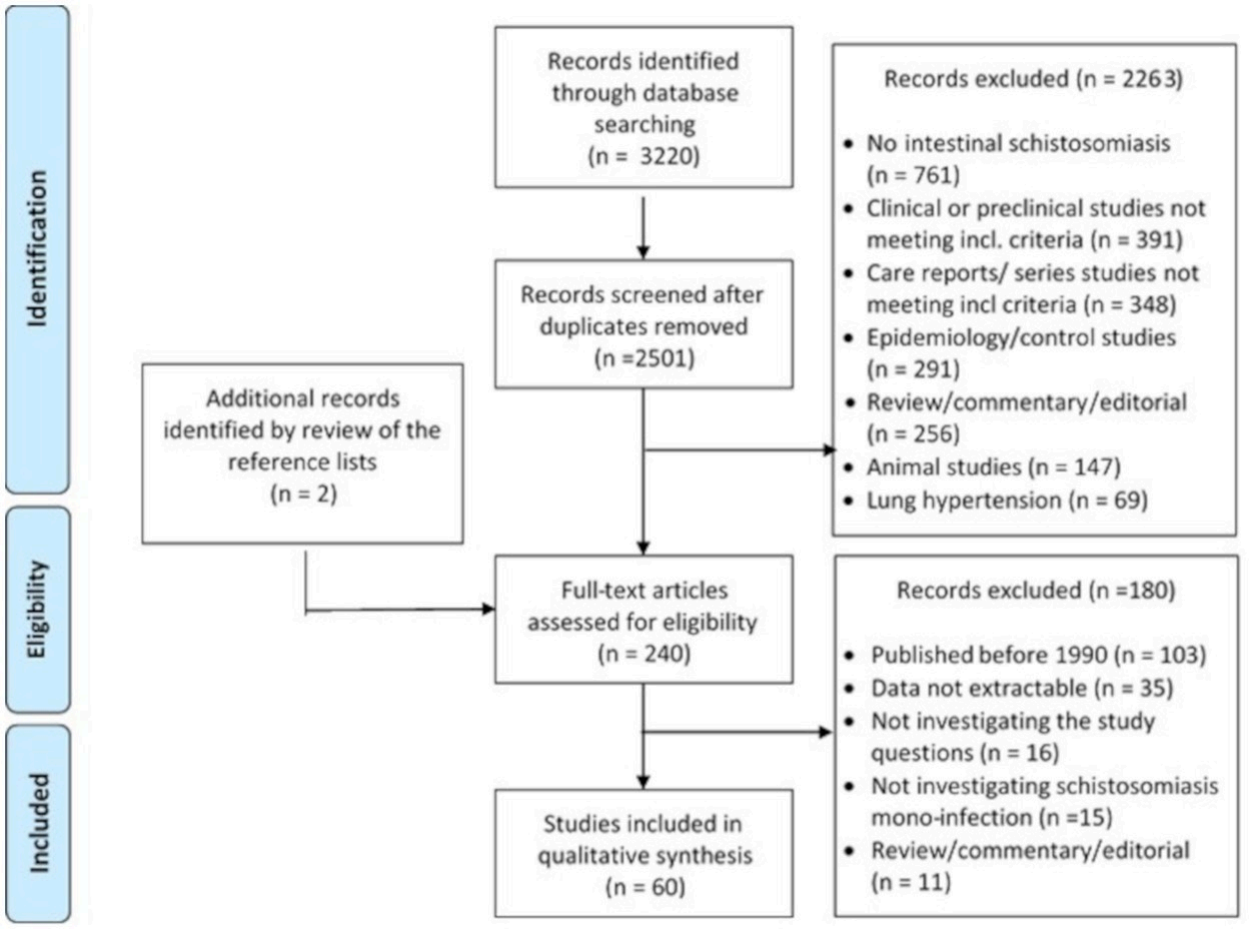
Literature search and selection of included studies.

The literature search and selection of included studies is shown in Fig 2. Of the finally included 60 publications, 13 focused on diagnostic aspects, 45 on therapeutic aspects, and 2 on both aspects. Full data extracted from the 60 included papers are available in S2 File. Although the actual species of *Schistosoma* was seldom specified in the publications, it can be assumed that *S*. *mansoni* was the involved species, with the exception of studies from China where *S*. *japonicum* is endemic [1].

### Diagnostic tools for clinical assessment

The definitive diagnosis and grading of upper gastrointestinal varices and the assessment of risk of bleeding are achieved by endoscopy. However, this relatively invasive technique is not always easy to implement in schistosomiasis endemic countries and is not always well accepted. Therefore, research has focused on the identification of noninvasive diagnostic parameters, mainly based on ultrasound but also on hematochemical parameters, to stratify patients according to the risk of presence of varices and bleeding, and, as the consequence, to decide who should undergo endoscopy and with what priority.

A brief summary of the 15 reviewed publications concerning the diagnostic parameters for the diagnosis and prognosis of the complications of HSS is presented in Table 2. Eight studies investigated the grade of PPF that, although heterogeneously classified, was found consistently associated with clinical parameters (presence and/or grade of varices and/or previous UGB). PPF grade is also included in the *S*. *mansoni* Score (SMS) [29], together with the portal vein (PV) diameter/height ratio. The SMS has been associated with presence of varices, variceal grade, occurrence of past episodes of UGB, and, most importantly, risk of future rebleeding episodes [29,30], Mohammed and colleagues [30] found that an SMS ≥2 was 95% sensitive for the presence of large varices, although poorly specific (58%), and a similar result can be extrapolated from the cohort described by Richter and colleagues [29]. Importantly, in this latter study, the highest risk of future variceal bleeding was associated with an SMS of 3 and 4 (odds ratio (OR), 144) compared to an SMS ≤2, and the number of bleeding episodes per 10 months during the follow-up increased from 0 (95% CI 0 to 0.013) for patients with an SMS <2 to 0.82 (95% CI 0.41 to 1.47) for SMS = 4.

**Table 2:** Summary of the included studies investigating diagnostic parameters for the diagnosis and prognosis of the complications of hepatosplenic schistosomiasis.

First author	Year	Country	Study design	N Sch patients	Grading system	Index technique	Principal findings
Abdel-Wahab [33]	1992	Egypt	Diagnostic accuracy	43	Thickness (in mm) of peripheral portal branches	Ultrasonography	History of hematemesis and grade of EV associated with grade of PPF
Richter [34]*	1992	Brazil and Sudan	Case–control	59	Qualitative grading of PPF	Ultrasonography	History of UGB associated with PV/height ratio and SV/height ratio; SV/height associated with presence of EV
Richter [35]*	1992	Brazil and Sudan	Case–control	59	Quantitative grading of PPF	Ultrasonography	Grade of PPF (2 and 3) associated with presences of EV and history of UGB
Abdel-Wahab [36]	1993	Egypt	Cohort	43	Thickness (in mm) of peripheral portal branches	Ultrasonography	US score (PPF grade, PV, spleen diameter, collaterals) associated with presence of EV and history of hematemesis
Domingues [37]	1993	Brazil	Cohort	105	Qualitative evaluation of PPF based on Homeida 1988 [38]	Ultrasonography	Grade of PPF (2 and 3) and history of UGB associated with presence and increasing grade of EV
Eltoum [39]	1994	Sudan	Case–control	257	Qualitative evaluation of PPF based on Homeida 1988 [38]	Ultrasonography	Grade of PPF (>1), SV (but not PV), spleen diameter and grade of EV associated with history of UGB
Richter [29]	1998	Brazil	Cross-sectional and cohort	50	Quantitative and qualitative grading of PPF	Ultrasonography	SMS (PPF grade, PV/height ratio) associated with history of UGB, EV grade, risk of rebleeding
Martins [40]	2000	Brazil	Cross-sectional	40	According to WHO Cairo 1991 [41]	Ultrasonography and Doppler	Pts with history of UBG had larger PV (but not SV) and PP thickness, and more frequent gastropathy and fundal varices
Maurizio [42]**	2000	Uganda	Cohort	34	Performed but not detailed in the abstract	Ultrasonography	Correlation between grade of PPF and grade of EV
Arruda [43]	2008	Brazil	Case–control	24	According to WHO Cairo 1991 [41]	Doppler ultrasonography	Pts with history of UGB had higher PV flow velocity but not different PV than patients without history of UGB
Ferreira [44]**	2009	Brazil	Cohort	146	None reported	Doppler ultrasonography	After EGDS pts with progression of EV have higher PV flow velocity at all follow-up time points
Agha [31]	2010	Saudi Arabia	Case–control	43	None reported	Platelet count/spleen diameter ratio	Platelet count (per ul)/spleen diameter (mm) ratio <885 had 100% sensitivity and 92% specificity for presence of EV
Ferraz [45]	2011	Brazil	Case–control	169	Fibrosis graded by histology	Ultrasonography	Pts with inversion of PV/SV ratio pre-EGDS have lower incidence of PV thrombosis but not rebleeding after EGDS
Xu [32]	2016	China	Cohort	95	None reported	Platelet count/spleen diameter ratio	Platelet count (per ul)/spleen diameter (mm) ratio <1,004 had 85% sensitivity and 83% specificity for presence of EV
Mohammed [30]	2018	Sudan	Cohort	100	Qualitative evaluation of PPF based on Homeida 1988 [38]	Ultrasonography and platelet count	SMS, platelet count, PV and PPF grade associated with presence of EV

EGDS, esophagogastric devascularization procedure with splenectomy; EV, esophageal varices; PPF, periportal fibrosis; Pts, patients; PV, portal vein diameter; Sch, schistosomiasis; SMS, *S*. *mansoni* score; SV, splenic vein diameter; UGB, upper gastrointestinal bleeding; US, ultrasound.

*Same patients’ cohort.

**Data extracted from abstract.

Other ultrasonographic (PV diameter, splenic vein (SV) diameter) and Doppler ultrasound parameters (PV and SV flow and flow velocity), as well as the spleen size, have been inconstantly associated with the abovementioned clinical parameters. However, the spleen size was measured in different ways in the included published literature (clinically or by ultrasound, bipolar diameter more or less normalized by the patient height, spleen volume), which makes results difficult to compare. Also, 2 out of 3 studies investigating the platelet count/spleen diameter ratio found high sensitivity (85% to 100%) and specificity (83% to 92%) for the presence of esophageal varices [31,32].

### Treatment approaches

Bleeding from esophageal varices is the most dramatic and dangerous consequence of portal hypertension in HSS. Multiple approaches are used to prevent it, most commonly applied after the first bleeding episode, while less data is available on primary prophylactic interventions. The most common treatment approaches include drug therapy with beta-blockers, variceal sclerotherapy or banding, esophagogastric devascularization with splenectomy (EGDS) techniques, and splenorenal shunting, while TIPS has been applied more recently.

### Pharmacological approach to portal hypertension with beta-blockers

**Fig 3:**
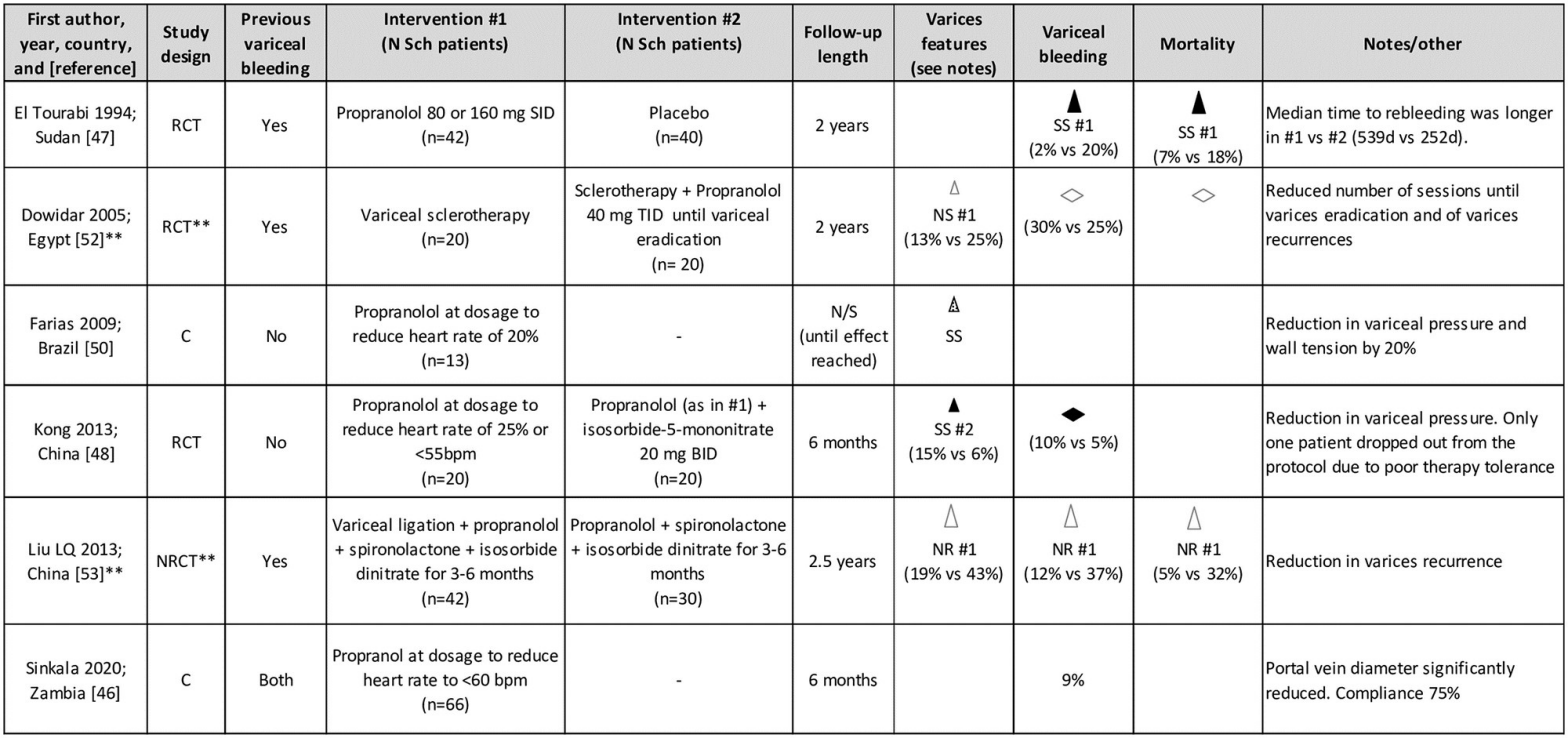
Direction effect chart summary of the included studies investigating the use of beta-blockers for the clinical management of hepatosplenic schistosomiasis. Evaluation of all outcomes refers to the end of follow-up. **Data extracted from abstract. Sch, schistosomiasis. C, Cohort study. C–C, Case–Control study. RCT, Randomized Clinical Trial. NRCT, Non-Randomized Clinical Trial. N/S, not specified. Triangle orientation indicates direction of outcome: upward = amelioration in respect to other intervention or baseline, horizontal = no difference between interventions or from baseline. Triangle size indicates sample size per (smallest) group: small ≤20 pts, medium 21–49 pts, large ≥50 pts. Triangle color indicates quality of result based on study design and source: black = RCT, light gray = C–C, dotted = C, white = data from abstract. SS, statistically significant, NS, not statistically significant, NR, statistical analysis not reported. #n = intervention indicated in the corresponding “Intervention #n” column of the table to which the outcome direction refers [46–48,50,52,53].

Six publications investigated the use of propranolol, a nonselective beta-blocker, in the clinical management of HSS (Fig 3). Propranolol was used for variable periods of time, from a few months to the entire length of follow-up (max 2 years in the included studies) at dosages ranging from 60 to 160 mg/day, until reaching the target effect of resting pulse rate reduced by 20% to 25% or <55 to 60 bpm. In no cases was PPF grading declared. In general, propranolol therapy was reported as well-tolerated; however, compliance was discussed as an issue by the majority of Authors. Sinkala and colleagues [46] reported a compliance of only 75% in their cohort. Propranolol was reported to reduce significantly the rate of rebleeding within 2 years compared to placebo (2% versus 20%) by El Tourabi and colleagues [47]; median time to rebleeding was also longer in the treatment compared to the placebo control group. Frequency of first-episode bleeding under propranolol treatment was reported at 5% by Kong and colleagues [48], although unfortunately, a nontreatment control group was not included in the study. A 9% bleeding rate within 6 months under propranolol was reported by Sinkala and colleagues [46], but also in this case, no control group was included and it was not possible to discern for what proportion of patients the bleeding episode was the first one or a recurrence. From a hemodynamic point of view, beta-blockage in patients with HSS appears to induce an increase in systemic resistance and a slight increase in pulmonary pressure, while significantly reducing the flow of all portal-afferent veins and of the Azygos vein [16,49]. Reduction in portal vein diameter upon treatment with propranolol was reported by Sinkala and colleagues [46], while no effect was observed on spleen size. Importantly, variceal pressure and wall tension reduction were observed by Farias and colleagues [50]. None of the retrieved studies addressed treatment with carvedilol, which has recently been discussed as a better treatment option compared to propranolol for the reduction of portal venous pressure in cirrhotic patients [51].

### Variceal management interventions

**Fig 4:**
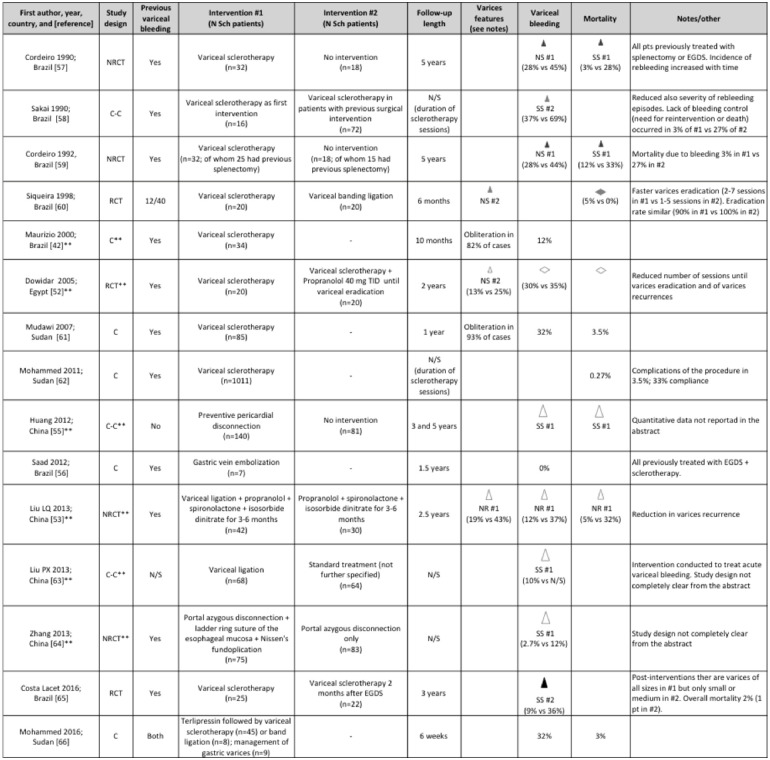
Direction effect chart summary of the included studies investigating the use of variceal management techniques for the clinical management of hepatosplenic schistosomiasis. Evaluation of all outcomes refers to the end of follow-up. **Data extracted from abstract. § Study involving 1,073 patients, 93% of whom with schistosomiasis. EGDS, esophagogastric devascularization procedure with splenectomy; N/S, not specified; Sch, schistosomiasis. C, Cohort study. C–C, Case–Control study. RCT, Randomized Clinical Trial. NRCT, Non-Randomized Clinical Trial. UGB, upper gastrointestinal bleeding. Triangle orientation indicates direction of outcome: upward = amelioration in respect to other intervention or baseline, horizontal = no difference between interventions or from baseline. Triangle size indicates sample size per (smallest) group: small ≤20 pts, medium 21–49 pts, large ≥50 pts. Triangle color indicates quality of result based on study design and source: black = RCT, dark gray = NRCT, light gray = C–C, dotted = C, white = data from abstract. SS, statistically significant, NS, not statistically significant, NR, statistical analysis not reported. #n = intervention indicated in the corresponding “Intervention #n” column of the table to which the outcome direction refers [42,52,53,55–66].

Sixteen publications included data on the outcome of variceal management, using sclerotherapy, band ligation, or other techniques. Only 1 publication [54] focused specifically on the management of gastric variceal bleeding, which was treated with N-butyl-cyanoacrilate injection in 100 patients; recurrent bleeding occurred in 12.5% cases within 24 h from the first intervention (controlled with further injection in 60% of these cases) and mean number of sessions for obliteration was 1.27. A summary of the remaining 15 publications is shown in Fig 4.

Although band ligation is currently the method of choice for the local treatment of esophageal varices, sclerotherapy was used in the vast majority of studies, possibly due to local constraints. In no case was local variceal management investigated specifically for primary prevention of bleeding. Granted the heterogeneity of follow-up lengths, the results of these studies indicate that sclerotherapy is followed by a high recurrence rate of varices and variceal bleeding, although lower than in case of no intervention. Recurrence was observed in about 30% of patients (from 10% to 69% depending on the publication), with increased incidence with time. These figures were roughly 50% to 75% lower when sclerotherapy was used after other interventions such as EGDS. Of note, many Authors discussed problems with adherence to the schedule of repeated sclerotherapy sessions required for this type of intervention.

Two included papers described interventions on veins feeding gastroesophageal varices (gastric, azygos) [55,56]. Although it is not possible to attempt any comparison of outcomes between these interventions and local variceal management techniques, the rate of rebleeding after portal-azygous disconnection was within the range of that reported in publications on local variceal management. Huang and colleagues [55] reported that the rate of first UGB episodes at 3 and 5 years was significantly lower, compared to no intervention, when preventive pericardial disconnection was applied on patients who had never bled before, but no numerical data were provided in the abstract. Saad and colleagues [56] found no rebleeding episodes, within 1.5 years from intervention, when gastric vein embolization was performed in patients treated with EGDS and variceal sclerotherapy but still unsatisfactory bleeding control.

### “Classic” surgical interventions

**Fig 5:**
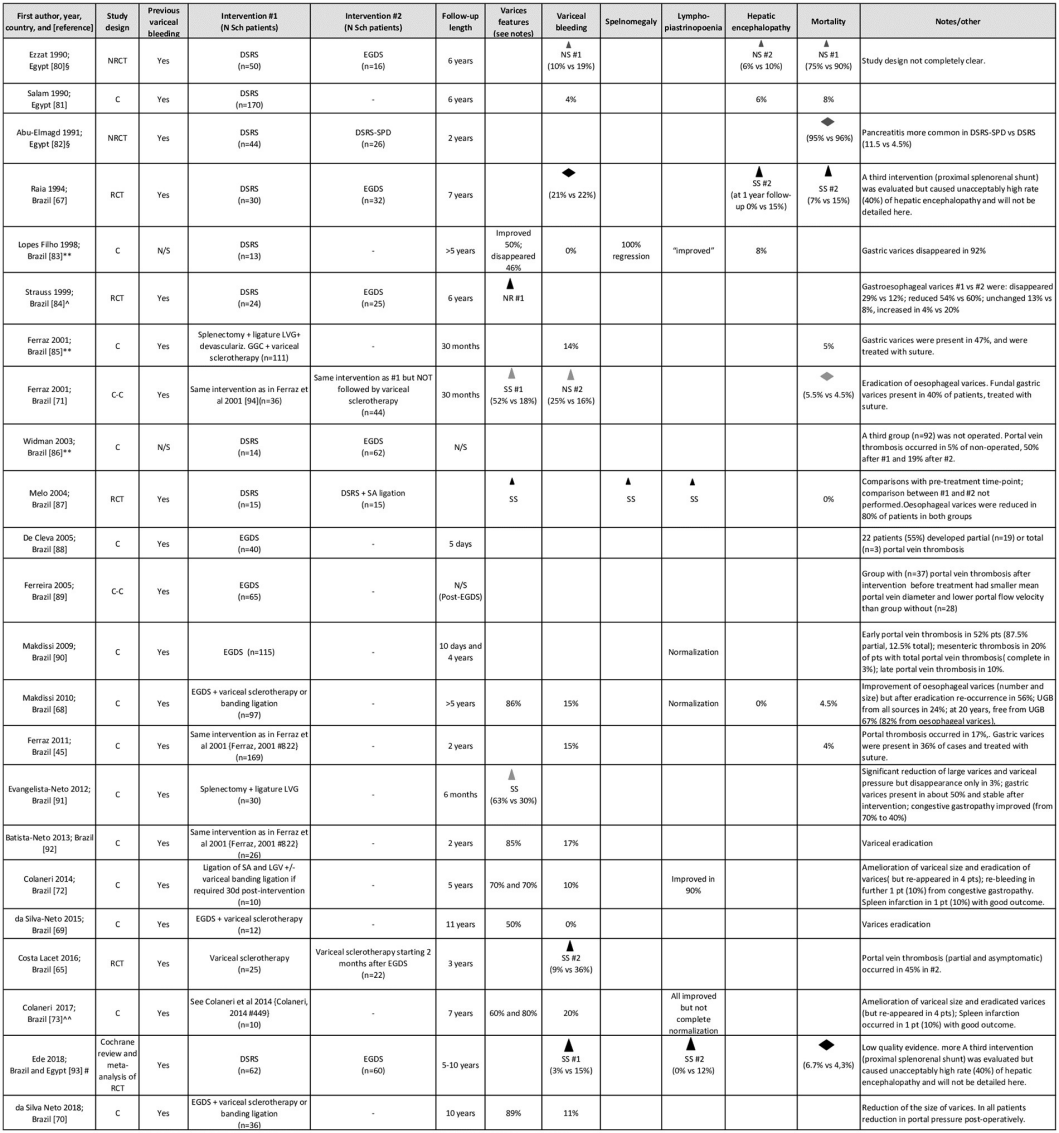
Direction effect chart summary of the included publications describing patients with hepatosplenic schistosomiasis treated with classic surgical interventions. Evaluation of all outcomes refers to the end of follow-up. DSRS, distal splenorenal shunt; DSRS-SPD, DSRS with splenopancreatic disconnection; EGDS, esophagogastric devascularization procedure with splenectomy; GGC, great gastric curvature; LGV, left gastric vein; N/S, not specified; SA, splenic artery; UGB, upper gastrointestinal bleeding. **Data extracted from abstract. §Study included also patients with other pathologies; as not all outcomes were compared between intervention groups in patients with schistosomiasis-only infection, principal findings refer to those parameters which could be extracted for this latter group. ^Subgroup analysis of the Raia and colleagues’ 1994 study. ^^Expanded follow-up of the cohort of Colaneri and colleagues’ 2014 study. #Cochrane review including 2 studies, Raia and colleagues [67] from Brazil and Gawish and colleagues [94] from Egypt (this latter study was retrieved by our literature search, but full text was unavailable). Column of the table. C, Cohort study. C–C, Case–Control study. RCT, Randomized Clinical Trial. NRCT, Non-Randomized Clinical Trial. UGB, upper gastrointestinal bleeding. Triangle orientation indicates direction of outcome: upward = amelioration in respect to other intervention or baseline, horizontal = no difference between interventions or from baseline. Triangle size indicates sample size per (smallest) group: small ≤20 pts, medium 21–49 pts, large ≥50 pts. Triangle color indicates quality of result based on study design and source: black = RCT, dark gray = NRCT, light gray = C–C, dotted = C, white = data from abstract. SS, statistically significant, NS, not statistically significant, NR, statistical analysis not reported. #n = intervention indicated in the corresponding “Intervention #n” to which the outcome direction refers [45,65,67–73,80–93].

The classic surgical management of HSS envisages selective portal-systemic shunts, mainly the distal splenorenal shunt (DSRS), and non-shunt interventions, mainly EGDS, and their variations, as synthesized in Fig 5. At present, none of these procedures appears clearly superior or to be preferred over the other, while proximal splenorenal shunt surgery is not recommended due to the unacceptably high rate of hepatic encephalopathy [67]. No study evaluated such surgical interventions in patients before the first bleeding episode.

Granted the heterogeneity of the reviewed studies for what concerns follow-up length and ancillary interventions (e.g., local management of varices), overall, DSRS appears being followed by fewer episodes of UGB compared to EGDS, but being burdened by a more frequent development of hepatic encephalopathy, with comparable mortality rates between the 2 interventions. When looking at the whole picture of results of the included studies presented in Fig 5, it seems that a better outcome in terms of rebleeding was reported when EGDS was followed by variceal sclerotherapy or band ligation [68–70], but no study included in this review formally compared EGDS alone versus EGDS followed by local variceal management. Such comparison was carried out by Ferraz and colleagues [71] who applied a variation of the EDGS procedure, including splenectomy, ligature of the left gastric vein, and devascularization of the great gastric curvature, without, however, finding significant differences in rebleeding rate between patients undergoing such technique followed or not by local variceal management sessions. Also, although not formally compared, the rebleeding rate after this variant intervention seems overall comparable with that following EGDS, and the Authors commented on the lower surgical complexity and risk of this variant procedure compared to the classic EGDS [71]. Among variant interventions, Colaneri and colleagues [72,73], aiming to preserve the spleen while performing vascular disconnection, applied a technique envisaging ligation of the splenic artery, ligation of the gastric vein, and variceal banding if required at endoscopic evaluation 30 days postintervention. This pilot study derived from their observation that during EGDS, the most important surgical moment, from the hemodynamical point of view, was the ligature of the splenic artery. Within the 7 years follow-up of the 10 included patients, good outcomes were reported, with only 1 patient developing spleen infarction, which resolved with conservative therapy [72,73].

Splenectomy is advocated to intervene both on portal hypertension caused by the passage of high-pressure blood from the arterial to the portal splenic circulation [74] and haematochemical abnormalities, mainly leukocytopenia and thrombocytopenia. However, the consequences of splenectomy in patients with schistosomiasis living in areas endemic for malaria have not been specifically addressed [75] as well as the risk for encapsulated bacterial infections. Furthermore, the role of the spleen in HSS, in particular for what concerns the etiology and clinical impact of thrombocytopenia, leukopenia, and coagulation, is not completely understood [10,12,76]. The group of Petroianu in Brazil [77–79] performed subtotal splenectomy in the context of EGDS procedure, reporting good outcomes in terms of both variceal regression and rebleeding, and hematochemical parameters recovery; however, spleen-preserving procedures are still not widely applied.

### Transjugular intrahepatic portosystemic shunt (TIPS)

In liver cirrhosis, TIPS has successfully been used since 1988 to reduce portal venous pressure and risk of recurrent bleeding [95]. TIPS is now a standard procedure in selected patient groups, where a survival benefit as well as improvement of quality of life has been shown [96,97].

In portal hypertension due to schistosomiasis, TIPS implantation has only rarely been reported, mostly in single-case reports or small case series. To our knowledge, the first cohort of patients with HSS treated with TIPS has been described by Dondelinger in 1997 [98], but only 6 out of the 48 described patients had pure schistosomal infection, and results of these specific subset of patients were not detailed. In commenting their experience, the Authors discussed about the technical difficulties in performing the TIPS procedure in these patients, and the very high frequency of reintervention within 2 years due to shunt dysfunction, which caused rebleeding. Four reports describing 25 patients with pure HSS treated with TIPS were included in this review (Table 3). This technique appears very promising, but so far, little data are available on the long-term outcome of such intervention, especially for what concerns the complications on the implant, the occurrence of rebleeding and hepatic encephalopathy, and the effect, if any, on splenomegaly/hypersplenism and hematochemical parameters. Dondelinger and colleagues [98] (not included in this review) reported a reduction in spleen size after TIPS implant in some patients, but splenomegaly persisted. Regarding the hepatic encephalopathy rate, this would appear lower than or comparable to that following DSRS; however, it is difficult to drive conclusions due to the small number of published cases managed with TIPS and the heterogeneity in the length of follow-up. The largest cohort of patients with HSS treated with TIPS was described from China [99] and reported the highest rate of development of portal encephalopathy, which in other published cases seems having occurred only in patients coinfected with viral hepatitis or precipitating factors [75,100]. It remains therefore to clarify if the peculiar clinical characteristics (e.g., refractory ascites) and apparently high rate of portal encephalopathy observed in the Chinese cohort were due to the presence of coinfections that were not diagnosed, or to a specific disease course due to infection with *S*. *japonicum* as opposed to *S*. *mansoni*.

**Table 3:** Summary of the included publications describing patients with hepatosplenic schistosomiasis treated with TIPS.

First author	Year	Country	N Sch patients	Previous variceal bleeding	Previous treatments	Length of follow-up	Principal findings
Richter [75]	2015	Germany (patient from Guinea)	1	Yes	Variceal band ligation	3 years	• Pre-TIPS, the patient had splenomegaly, thrombocytopenia, leukopenia, anaemia, increased INR and PTT, and large varices. • Post-TIPS, there was regression of grade 1 varices; no information on spleen size and hematological parameters are provided. • One episode of hepatic encephalopathy occurred, concomitant to acute viral gastroenteritis. • TIPS needed revision due to displacement after 10 months.
Grieco [102]	2016	Italy (patient from Ethiopia)	1	Yes	Variceal band ligation	Not reported	• Pre-TIPS, the patient has splenomegaly, thrombocytopenia, leukopenia, anaemia, slightly increased INR, and moderate ascites. • Post-TIPS, there was no immediate episode of hepatic encephalopathy. • No follow-up outcome data are reported.
Kraef [100]	2019	Germany (patient from Philippines, Sierra Leone, and Eritrea)	3	Patient 1 –Yes (unclear HBV coinfection)	Variceal band ligation + carvedilol	9 months	• Pre-TIPS, the patients had grade II/III varices. • Post-TIPS, no further bleeding and occurrence of 2 episodes of mild hepatic encephalopathy. • No information on spleen size and haematochemical parameters.
				Patient 2 –Yes	Variceal band ligation	24 months	• Pre-TIPS, the patient had splenomegaly and pancytopenia and grade III varices. • Post-TIPS, regression of varices, no episodes of rebleeding and no episodes of hepatic encephalopathy were observed; no changes of the spleen size or hematological parameters
				Patient 3 –No	Variceal band ligation	48 months	• Pre-TIPS, there was massive splenomegaly, pancytopenia, grade II varices • Post-TIPS, there was no episode of rebleeding and no episode of hepatic encephalopathy; no information on varices or spleen size, or hematological parameters
Liu J [99]	2019	China	20	Yes (*n =* 16); No but refractory ascites (*n =* 4)	Variceal band ligation (n = 9); EGDS (*n* = 12)	Mean 15 months (2–30)	• Pre-TIPS, in those with UGB, in 7 bleeding was from esophageal and in 9 from gastric varices; these were embolized during TIPS procedure; mean spleen size increased; mean prothrombin time moderately increased, mean platelet count normal • Post-TIPS, hepatic encephalopathy occurred in 25% (5/20) of patients, UGB in 15% (3/20; not specified if from esophageal or gastric varices); mortality was 15% (3/20; *n* = 1 from renal failure; *n* = 2 from UGB)

EGDS, esophagogastric devascularization procedure with splenectomy; INR, intentional normalized ratio; PTT, partial thromboplastin time; Sch, schistosomiasis; TIPS, transjugular intrahepatic portosystemic shunt; UGB, upper gastrointestinal bleeding.

### Liver transplantation

As liver function is generally preserved in HSS, liver transplantation is not frequently considered a needed treatment option. In our literature search, we found only 1 cohort study from Saudi Arabia [101] including 11 patients with HSS and 3 patients with HSS associated with viral hepatitis who received right lobe liver grafts from live donors (*n =* 5) or whole liver grafts from deceased donors (*n* = 9) because of variceal bleeding and/or ascites and/or portal vein thrombosis. Reported survival at 1 and 5 years was 75%, with 3 patients dying within the first year posttransplant, only 2 of them for reasons related to the intervention (sepsis and disseminated intravascular coagulation). After transplantation, neither splenomegaly nor thrombocytopenia improved.

## Discussion

In recent years, there has been considerable effort by the international community to decrease morbidity due to schistosomiasis with mass drug administration of praziquantel [103]. While this strategy is effective in reducing the burden of infection and pathology in communities, still a proportion of individuals will develop chronic, irreversible complications of schistosomiasis due to poor or late access to medical treatment [104]. Indeed, interruption of fibrosis progression and partial or complete regression of fibrosis and portal hypertension after months from praziquantel treatment has been reported in several studies [18,19]; however, this is observed mostly in the presence of early-grade fibrosis and in patients of young age. In this scoping review, we present the state of knowledge on current options for the management of HSS to provide a comprehensive picture of consolidated findings and knowledge gaps and help define currently available clinical management strategies and the future lines of clinical research.

HSS is a heterogeneous condition, ranging from mildly symptomatic to life-threatening disease. The development of HSS may be slow and with a few symptoms until the first episode of UGB occurs; furthermore, very advanced HSS can progress to liver failure [7]. It is therefore intuitive that no “one-size-fits-all” approach is likely to be appropriate for all clinical conditions occurring in HSS.

In addition, not just the pathological condition and the type of intervention per se but also the healthcare setting and expertise and the peculiar clinical and social conditions of the patient (e.g., the possibility or willingness to comply with a long-term follow-up, living setting, other potential infectious disease exposures) are relevant in deciding the best management approach for each patient. Currently, however, there is little evidence-based data on which the treating physician can rely upon for such a decision.

The haemodynamic alterations at the basis of portal hypertension in HSS are still not completely understood and likely result from more than one mechanism [13,14]. Furthermore, knowing the occurrence or the predominance of a mechanism over the other(s) may be important to guide the most appropriate clinical management. El-Gendi and colleagues [14] suggested using the comparative measurement of the portal pressure through the cannulated umbilical vein and splenic vein to decide whether presinusoidal hypertension or splenic hyperafflux predominated, and therefore what intervention could best fit each patient. Unfortunately, to our knowledge, this approach was not systematically undertaken, and there is currently no noninvasive way of measuring such venous pressures. Recently, Jeong and colleagues [105] explored the use of contrast-enhanced ultrasonography to estimate portal hypertension in cirrhotic patients with good results; a similar exploration, targeting both liver and spleen perfusions and their relationship, would be interesting in HSS. Also, recently, spleen stiffness measured by transient elastography was individuated as a potential surrogate marker of portal hypertension in patients with both cirrhosis and HSS; it would therefore be worth exploring further the potentials of this technique for the stratification of patients based on the risk of UGB and to guide clinical decision-making [106]. Even when using easily implementable tools such as ultrasonography, parameters (e.g., spleen size) have been measured and reported in very heterogeneous manners, and no patient stratification (e.g., based on grade of PPF according the current reference WHO classification [107]) was generally applied. This makes extremely difficult to compare the results of different studies. The score SMS was reported as very promising to stratify patients based on the risk of variceal bleeding [29]. Unfortunately, the SMS appears rarely applied in published studies and it should be updated by changing the numerical PPF grade with the A-F fibrosis pattern currently recommended for the grading of PPF in schistosomiasis [107,108].

When dealing with treatment options, local interventions on esophageal varices, such as sclerotherapy and band ligation, as the sole treatment, seem burdened by a high rate of rebleeding, and compliance with repeated intervention sessions and follow-up visits may be an issue. The same problem with compliance with long-term regular medication seems valid also for treatment with beta-blockers; possibly, the use of these drugs for a short and defined period of time such as that between first episode of bleeding and surgery, as suggested by Raia and colleagues [109], may result in better compliance.

Surgical approaches for HSS have the advantage of being somehow “definitive.” The 2 main approaches currently in use are selective portal-systemic shunts, mainly DSRS, and non-shunt interventions, mainly EGDS, and their variations. Besides technical difficulty and local expertise, which may induce to choose an intervention over another in different settings, the short/midterm overall outcome of these interventions seems comparable, with generally lower rate of UGB and portal vein thrombosis but higher rate of portal encephalopathy after DSRS compared to EGDS [93]. Preference for the application of EGDS over DSRS was expressed by several authors [67,110,111], but the opposite also by others [80,112].

Interestingly, a few publications described less invasive variations of EGDS, preserving or not the spleen [71,72,91], with seemingly good results. Such approaches may deserve further evaluation, as they may be more suitable in some less experienced or less equipped settings. Genzini and colleagues [113], in their review, reported the results of the study by Cury and colleagues [114], which unfortunately could not be retrieved. Reported results for this study included the comparison of rebleeding rate at 4 years after DSRS and after sclerotherapy, which was 7% and 33%, respectively. However, the authors also reported that only 16% of patients who could undergo repeated sclerotherapy eventually needed a surgical (i.e., more dangerous) intervention. These data stress even more the concept that not just the pathological condition and the type of intervention per se [93] but also the whole clinical setting (healthcare setting and expertise, environmental and patients’ personal living conditions and compliance) are relevant in deciding the best management approach for each patient.

The issue of spleen preservation or removal is a matter of debate. Splenectomy is advocated because the spleen contributes in these patients to the development and maintenance of portal hypertension and pancytopenia. However, the real need for total splenectomy has been questioned considering the apparently limited clinical relevance of coagulation and immunological alterations in HSS and the infection risks following this procedure [79]. Partial splenectomy or spleen autotransplantation with the peritoneal implant of spleen portions following total splenectomy are performed by some groups [77–79,115–117] but are not widely practiced. While the higher risk of infection with capsulated bacteria after splenectomy can be reduced by vaccinations, that of malaria is of concern [118], especially because the vast majority patients with HSS (who come from sub-Saharan Africa), will be exposed to malaria after the procedure.

TIPS is among the least explored interventions in patients with HSS. This technique appears very promising due to the relatively modest invasiveness, preservation of the spleen, and efficacy in inducing regression of esophageal varices at least in the short/midterm. However, some authors have discussed the technical difficulty in performing this procedure in patients with PPF and the high rate of complications requiring stent revision [98], which in resource-poor areas may not always be easily implemented. In liver cirrhosis, portal hypertension is only one life-limiting factor alongside with reduced liver function and increased risk of hepatocellular carcinoma. Therefore, TIPS is mostly used as a “bridge” intervention before transplant. On the contrary, in HSS liver function is usually preserved, which renders TIPS a potentially definitive treatment. However, there is still no data on its long-term outcome. In patients with cirrhosis, primary patency of stents has been shown in more than 60% for periods of time exceeding a decade with no drop of patency after the first 6 years [96]. Improved postprocedural stent thrombosis and long-time survival following TIPS has been associated with preserved liver function and with use of covered stents [119], which have only been introduced in the early 2000s [120]. The major late complication is occlusion or stenosis of the TIPS or the draining liver vein, which results in recurrent portal hypertension, and is usually addressed with reintervention (balloon dilation or positioning of another stent inside the original stent) or, if diagnosed early, with medical thrombolysis [121]. Future studies will have to address whether these observations and results also apply to patients with HSS.

In general, the follow-up of patients reported in the reviewed publications rarely exceeded the 5 years. Considering that patients with HSS are generally young, otherwise healthy adults, the absence of data regarding the long-term (ideally life-long) safety and efficacy of interventions is a problem, as it is the virtual absence of data on the use and outcome of different management approaches for the primary prevention of UGB.

Finally, the pathology caused by *S*. *mansoni* and *S*. *japonicum* have distinct features, and it would have been important to describe and compare the diagnostic and management strategies for the 2 infections. However, virtually never the species infecting the patients included in the reviewed studies was openly declared. Therefore, we could just assume that all infections were due to *S*. *mansoni* with the exception of the Chinese cohorts, which were most likely due to *S*. *japonicum*.

This study has several limitations. Firstly, we did not attempt performing a comparison of interventions using meta-analysis, and we did not formally assess the quality of the included publications. Also, due to our aim of providing a picture of currently available management options, we arbitrarily included only papers published from 1990; our rationale was that “old,” consolidated techniques still in use would have been used to compare the efficacy of newer, less consolidated techniques, and therefore described and evaluated also in more recent literature. We extracted data, whenever possible, also from abstracts, which provide a limited depth of understanding of the whole study. As a consequence, it is possible that interventions described in lower-quality studies have been given similar attention to more consolidated interventions, the results of which are supported by more/higher-quality studies. In part, this was balanced by the presentation of data from all individual publications in summary tables, which allows an appraisal of the study design. Furthermore, in accordance with the AMSTAR checklist to assess the quality of systematic reviews [122] and with the Cochrane Handbook for Systematic Reviews of Interventions [123] in defining the database search and the exploration of the gray literature, we searched the only 2 databases of peer-reviewed publications considered mandatory, and this may have excluded some potentially eligible publications. In addition, a number of potentially eligible studies could not be included because neither the abstract nor the full text could be retrieved, and this may have limited, as an example, the extent of the overview of studies on some techniques available from different geographical areas. For example, one could assume from Fig 5 that Chinese (supposing *S*. *japonicum* infected) patients are not managed by EDGS or DSRS. On the contrary, we retrieved 6 potentially eligible papers by title from China describing the use of these techniques, but we could not include them in our review, as data were not available for extraction [124–128]. Lastly, we did not discriminate between treatments performed in adults and in children/adolescents.

In conclusion, an overall appraisal of the reviewed literature shows that (Box 1 and Box 2): (1) most interventions have been developed in endemic areas on the basis of individual groups’ experiences and almost never rigorously compared; (2) there is a lack of data regarding which parameters can guide the choice of intervention and on which clinicians can base clinical management decisions; (3) there is a general lack of data on the long-term outcome of interventions, likely due to the extreme difficulty in carry out long-term, rigorous follow-up in endemic areas and, outside endemic areas, on patient populations basically constituted by highly mobile migrants; and (4) virtually no data exist on the application of interventions for primary prophylaxis purposes, i.e., to prevent the first episode of variceal bleeding. This overall picture highlights a dramatic need for the implementation of rigorous prospective studies in different settings, using shared and agreed definitions and procedures, to fill such fundamental gaps, still present for a disease affecting millions of patients worldwide. Furthermore, 2 further aspects of this complex disease surely deserve a focused, thorough investigation, i.e., the presence of coinfection with hepatitis viruses or other comorbidities, and the different presentation of pathology between children/adolescents and adults. Regarding the former, in medical practice in endemic areas, schistosomal liver fibrosis has a particularly severe outcome in patients with concomitant liver diseases such as viral hepatitis and/or toxic liver cirrhosis; this condition of comorbidity surely deserves attention and a targeted research aiming to evaluate similarities and differences with monoinfection and the state of the art of clinical management. Regarding the latter, it has been shown that children infected with schistosomiasis may present advanced pathology with hepatosplenomegaly, portal hypertension, and even varices, without detectable periportal fibrosis [129]. Different pathophysiological mechanisms [129], and therefore possibly different treatment approaches required, in children compared to adults, deserve investigation, especially in the light of the fact that praziquantel treatment is more effective in inducing pathology regression in early pathology and young patients [18,19,104].

**Box 1. Key learning points**

- Hepatosplenic schistosomiasis is a complex clinical condition with pathophysiological characteristics different from hepatic cirrhosis.
- The most common diagnostic approaches to stratify patients based on the risk of variceal bleeding include the use of ultrasonography and platelet counts.
- Therapeutic options include medical and interventional approaches, but most interventions have been developed on the basis of individual groups’ experiences and almost never rigorously compared
- There is little evidence-based data on which parameters can guide the choice of intervention for individual patients.
- Praziquantel must be administered to all patients with schistosomiasis but is often not sufficient to control and treat hepatosplenic schistosomiasis.

**Box 2. Top five papers**

1. WHO/Special Programme for Research & Training in Tropical Diseases. Ultrasound in schistosomiasis: a practical guide to the standard use of ultrasonography for assessment of schistosomiasis-related morbidity. Second international workshop, October 22–26 1996, Niamey, Niger. Editors: J. Richter, C. Hatz, G. Campagne, N. R. Bergquist, J. M. Jenkins. Number of pages: vii; 49 p. Publication date: May 2000. WHO reference number: TDR/STR/SCH/00.1.
2. Ede CJ, Nikolova D, Brand M. Surgical portosystemic shunts versus devascularisation procedures for prevention of variceal rebleeding in people with hepatosplenic schistosomiasis. Cochrane Syst Rev. 2018;8:CD011717.
3. Richter J, Correia Dacal AR, Vergetti Siqueira JG, Poggensee G, Mannsmann U, Deelder A, et al. Sonographic prediction of variceal bleeding in patients with liver fibrosis due to *Schistosoma mansoni*. Trop Med Int Health. 1998;3(9):728–735.
4. El-Gendi MA, Gemeuh N. Contrasting haemodynamic patterns of portal hypertension in hepatosplenic schistosomiasis. Lymphology. 1977;10(4):209–215.
5. Richter J, Bode JG, Blondin D, Kircheis G, Kubitz R, Holtfreter MC, et al. Severe liver fibrosis caused by *Schistosoma mansoni*: management and treatment with a transjugular intrahepatic portosystemic shunt. Lancet Infect Dis. 2015;15(6):731–737.

## Supporting information

S1 File Literature search strategy. (DOCX)

S2 File Original data file. (XLSX)

S1 Table PRISMA checklist. (DOCX)
