# Supplementary material for: Diagnosis and clinical management of hepatosplenic schistosomiasis: A scoping review of the literature
Source: PLoS Negl Trop Dis. 2021 Mar 25;15(3):e0009191. doi: 10.1371/journal.pntd.0009191 (PMC7993612; doi:10.1371/journal.pntd.0009191)
Supplement: S1 File — (DOCX) [file pntd.0009191.s001.docx]

**MEDLINE (PubMed) (1946 to 13 March 2020)**

(((((((((((((((((((((((("Schistosomiasis"[Mesh]) OR "Schistosomiasis mansoni"[Mesh]) OR "Schistosoma japonicum"[Mesh])) OR (Schistosomiases[Title/Abstract] OR "Schistoma Infection"[Title/Abstract] OR "Schistoma Infections"[Title/Abstract] OR "Katayama Fever"[Title/Abstract] OR Bilharziasis[Title/Abstract] OR Bilharziase[Title/Abstract] OR "Schistosoma mansoni Infection"[Title/Abstract] OR "Schistosoma mansoni Infections"[Title/Abstract] OR "intestinal Schistosomiases"[Title/Abstract] OR "Intestinal Schistosomiasis"[Title/Abstract] OR "Schistosoma japonicums"[Title/Abstract] OR "Schistosoma japonicum"[Title/Abstract] OR "schistosoma mekongi"[Title/Abstract]))))) OR schistosom*[Title/Abstract])))) OR (‘Hepatosplenic schistosomiasis’[Title/Abstract] OR ‘Hepatic schistosomiasis’[Title/Abstract] OR ‘Intestinal schistosomiasis’ [Title/Abstract] OR 'gastrointestinal schistosomiasis’[Title/Abstract])))))))) AND ((((((("Hypertension, Portal"[Mesh]) OR "Splenomegaly"[Mesh]) OR "Esophageal and Gastric Varices"[Mesh]) OR "Gastrointestinal Hemorrhage"[Mesh]) OR "Hemorrhage"[Mesh])) OR (splenomegaly[Title/Abstract] OR ‘oesophageal varices’[Title/Abstract] OR ‘gastro-oesophageal varices’[Title/Abstract] OR ‘gastrointestinal bleeding’[Title/Abstract] OR bleeding[Title/Abstract] OR ‘variaceal bleeding’[Title/Abstract] OR haemorrage[Title/Abstract] OR ‘Portal Hypertension’[Title/Abstract] OR ‘Portal Hypertensions’[Title/Abstract] OR ‘Cruveilhier-Baumgarten Syndrome’[Title/Abstract] OR ‘Cruveilhier Baumgarten Syndrome’[Title/Abstract] OR ‘Enlarged Spleen’[Title/Abstract] OR ‘Gastric Varix’[Title/Abstract] OR ‘Gastric Varices’[Title/Abstract] OR ‘Esophageal Varices’[Title/Abstract] OR ‘Esophageal Varix’[Title/Abstract] OR ‘Hemorrhage, Gastrointestinal’[Title/Abstract] OR ‘Gastrointestinal Hemorrhages’[Title/Abstract] OR Hematochezia[Title/Abstract] OR Hematochezias[Title/Abstract] OR Hemorrhages[Title/Abstract] OR Bleeding[Title/Abstract]))

**Embase (1974 to 13 March 2020)**

#1 'schistosomiasis'/exp OR 'schistosomiasis'

#2 schistosomiasi OR schistosomiasis OR schistosoma OR 'schistosomiasis haematobia' OR 'schistosomiasis japonica' OR 'schistosomiasis mansoni':ti,ab

#3 #1 OR #2

#4 schistosomiases OR 'schistoma infection' OR 'schistoma infections' OR 'katayama fever' OR bilharziasis OR bilharziase OR 'schistosoma mansoni infection' OR 'schistosoma mansoni infections' OR 'intestinal schistosomiases' OR 'schistosoma japonicums' OR 'schistosoma japonicum' OR 'schistosoma mekongi' OR schistosom* OR 'hepatosplenic schistosomiasis' OR 'hepatic schistosomiasis' OR 'intestinal schistosomiasis' OR 'gastrointestinal schistosomiasis':ti,ab

#5 #3 OR #4

#6 'portal hypertension'/exp/mj OR 'splenomegaly'/exp/mj OR 'esophagus varices'/exp/mj OR 'gastrointestinal hemorrhage'/exp/mj OR 'bleeding'/exp/mj

#7 splenomegaly OR 'oesophageal varices' OR 'gastro-oesophageal varices' OR 'gastrointestinal bleeding' OR bleeding OR 'variaceal bleeding' OR haemorrage OR 'portal hypertension' OR 'portal hypertensions' OR 'cruveilhier-baumgarten syndrome' OR 'cruveilhier baumgarten syndrome' OR 'enlarged spleen' OR 'gastric varix' OR 'gastric varices' OR 'esophageal varices' OR 'esophageal varix' OR 'hemorrhage, gastrointestinal' OR 'gastrointestinal hemorrhages' OR hematochezia OR hematochezias OR hemorrhages OR bleeding:ti,ab

#8 #6 OR #7

#9 #5 AND #8

#10 #5 AND #8 AND [embase]/lim
